# Eliciting psychiatric nurses’ preferences for workplace violence prevention: a protocol for discrete choice experiment

**DOI:** 10.3389/fpubh.2024.1296525

**Published:** 2024-07-03

**Authors:** Peng Xie, Hui-qin Li, Li Tao, Hao Yang

**Affiliations:** ^1^People's Hospital of Deyang City, Deyang, China; ^2^West China Hospital, Sichuan University/West China School of Nursing, Sichuan University, Chengdu, China

**Keywords:** workplace violence, psychiatric nurses, nursing management, discrete choice experiment, protocol

## Abstract

**Introduction:**

Workplace violence against healthcare workers has become a serious global public health problem. The incidence of workplace violence towards Psychiatric nurses is higher than in all other medical institutions, up to 84.2% per year. It not only negatively affects many aspects of healthcare workers’ lives, but also destroys the harmony of the nurse–patient relationship and reduces the quality of nursing care. The number of psychiatric nurses in China was approximately 96,000, far lower than most other countries and unable to meet the growing demand for mental health. However, the increase in workplace violence has future exacerbates the current shortage of nurses. Therefore, it is necessary to develop effective strategies to prevent psychiatric nurses from suffering from workplace violence, thereby to reduce nurse turnover and improve the quality of nursing care. A comprehensive understanding of psychiatric nurses’ preferences and priorities for preventing workplace violence is an important prerequisite before formulating strategies and taking measures. Unfortunately, to date, no research has investigated the psychiatric nurses’ preferences. Therefore, a discrete choice experiment (DCE) is conducting to explore the psychiatric nurses’ preferences for workplace violence prevention. This article reports on methodological details of the DCE.

**Methods and analysis:**

Six attributes were developed through a literature review, one-on-one interviews and focus group discussions. D-efficient design in NGENE was used to generate choice sets. SPSS 24.0 will be used for descriptive analysis of social Demography, and Stata 16.0 will be used for analysis of DCE data. A multinomial logit model will be used to preliminarily explore trade-offs between workplace violence prevention characteristics included in the choice tasks. Then, in a mixed logit model, we plan to choose some arbitrarily defined base violence prevention program and will use the nlcom command to evaluate the probability of an alternative violence prevention program.

**Ethics and dissemination:**

The study was approved by the relevant ethics committees. Our findings will emphasize priority intervention areas based on the preferences of psychiatric nurses and provide references for hospitals to develop and improve workplace violence prevention strategies. The results will be shared through seminars, policy briefs, peer-reviewed journal articles and online blogs.

## Introduction

Workplace violence is defined as “the act or threat of violence, ranging from verbal abuse to physical assaults directed toward persons at work or on duty” by The National Institute for Occupational Safety and Health (1). The most common forms of workplace violence are physical and verbal attacks (2). Workplace violence against healthcare workers is a common occurrence in healthcare environments in many countries, and has become a serious global public health problem (2). In psychiatry departments or psychiatry hospitals, the incidence of workplace violence towards nurses is higher than in all other medical institutions (3, 4), with 84.2% experiencing workplace language and/or physical violence within 1 year (5).

The reasons for this phenomenon are multifaceted. One study confirmed that the risk of violence from people with mental disorders is significantly higher than that from people without mental disorders (6). Meanwhile, a review reiterated that almost one fifth of acute psychiatric patients engage in violent behavior (7). Psychiatric nurses come into face-to-face contact with patients with mental instability or mood disorders, so they are at higher risk of suffering workplace violence (3, 5), which leads to high psychological stress for nurses, and the accumulated stress can lead to high levels of mood swings and losing their temper (8, 9). In addition, psychiatric nurses may have difficulty controlling emotions when facing violence. When patients become violent, nurses may also engage in aggressive behavior, which to some extent affects the nurse patient relationship and escalates violence (8). China is a developing country, and there is a gap in the level of its healthcare service system compared to developed countries. The limitations of medical technology and the shortage of high-quality medical resources make it difficult for mental illness patients and their families to achieve the expected recovery, which makes patients and their families more likely to have conflicts with nurses and doctors (10). The characteristics of supportive environment, including the environment of inpatients, the optimization of architecture and interior design, can reduce the incidence of psychiatric workplace violence (11). However, the low level of economic development in China is not enough to fully support the optimization of ward environment, which hinders the reduction and prevention of psychiatric workplace violence.

Many researches have confirmed that workplace violence not only negatively affects many aspects of healthcare workers’ lives, including emotional, psychological and physical, but also destroys the harmony of the nurse–patient relationship and reduces the quality of nursing care (12–14). The global burden of mental disorders increased by 13.5% from 2007 to 2017, which has become a global public health problem that needs to be addressed urgently (15, 16). However, there is still a serious shortage of psychiatric workforce (17, 18). In 2019, the number of nurses in psychiatric hospitals/departments in China was approximately 96,000, far lower than most other countries and unable to meet the growing demand for mental health (19, 20). The increase in workplace violence has become an important factor in the turnover of nurses, which affects the stability of nursing teams and further exacerbates the current shortage of nurses (21, 22). Therefore, it is necessary to develop effective strategies to prevent psychiatric nurses from suffering from workplace violence, so as to reduce nurse turnover and improve the quality of nursing care.

Many studies have explored prevention and response measures for workplace violence (23–28), but these studies are mostly conducted in developed countries, and there are no quantitative research results targeting the views of psychiatric nurses. Although many measures have been taken to reduce workplace violence (29, 30), but their effectiveness has not been satisfactory, and workplace violence against healthcare workers remains an urgent issue to be addressed in China (31–33). Preventive strategies developed based on the needs and preferences of psychiatric nurses are more effective. Although few qualitative studies have focused on the influencing factors of psychiatric nurses suffering from workplace violence and their views on prevention of workplace violence (8, 11, 34). Unfortunately, due to the inherent limitations of qualitative research, the relative importance of these factors cannot be weighed, and no study has validated the effectiveness of preventive measures proposed by psychiatric nurses. This provides very limited reference for developing effective strategies to prevent workplace violence among psychiatric nurses. In addition, mass media reports of violent crimes committed by mentally ill people against health professionals worldwide have caused public anger and anxiety, which has made the public aware of the need to create safer working environments for health professionals and to develop and strengthen appropriate laws and regulations to prevent workplace violence (35). A comprehensive understanding of psychiatric nurses’ preferences and priorities for preventing workplace violence is an important prerequisite before taking measures and formulating laws and regulations (36). However, no study to date has explored psychiatric nurses’ preferences and their priorities for workplace violence prevention.

Patients’ preferences are inner experiences, which cannot be quantified using traditional survey research and qualitative research. Discrete choice experiment (DCE) is a preference measurement method, which overcomes the limitation that traditional survey research and qualitative research cannot quantify preferences. This method has formed a relatively complete theoretical system, which combines the consumption demand theory (37), econometric analysis (38), random utility theory (39) and experimental design theory (40), and is increasingly used in the field of health care. In a DCE study, it is assumed that the characteristics of an intervention (policy or service) can be described by the corresponding attributes and levels, and the preferences of the individuals are described by their trade-offs between the options composed of attributes and levels, thus quantifying the preferences of the individuals and the priorities of the measures and or policies (41–43).DCE achieves the following values by assessing the strength of preferences: (1) collecting a large amount of relevant data at a moderate cost to optimize resource allocation; (2) providing references for the formulation of policies (programs or services); and (3) Providing data for economic evaluation and decision making (42–44). Conducting a DCE to explore the preferences of psychiatric nurses for preventing workplace violence can provide reference for formulating and optimizing laws and regulations to prevent workplace violence, thereby reducing the occurrence of workplace violence, reducing nurse turnover, and improving nursing quality. This manuscript describes the protocol for the ongoing DCE study.

## Method

### Study setting

This study has been conducting in the southwestern region of China, with plans to collect data from seven hospitals in the region, including four general hospitals and three psychiatric hospitals, all of which have well-established psychiatric care systems and psychiatric nurses.

### Study design

This is a cross-sectional study amongst psychiatric nurses using a survey that includes the Discrete Choice Experiment (DCE). Psychiatric nurses will complete this paper-and-pencil survey under the guidance from a research assistant. The design and execution of this study followed the principle of discrete choice experimental design, taking the following steps: (1) Identifying attributes and defining levels; (2) Generating choice sets; (3) Questionnaire design and pilot testing; (4) Samples and recruitment; and (5) Data analysis.

### Identifying attributes and defining levels

Multiple choice tasks compose a DCE, and respondents are asked to choose between two (or more) hypothetical violence prevention programs. A key element in the design of the DCE is to identify the key features (attributes) that describe the violence prevention programs in the choice tasks. The attributes and levels included in this study were developed in several phases.

#### Literature review

A comprehensive review of literature can help identify potential attributes and provide reference for qualitative research. We have searched the literatures in databases such as Pubmed, Cinahl, Embase, Wanfang, and CNKI. In order to obtain more comprehensive information, we have used a snowball method to review the references of the retrieved literature.

#### One-on-one interviews

To ensure sufficient heterogeneity, a purposive sampling has been conducted on psychiatric nurses who met the inclusion and exclusion criteria based on seniority, education level, type of hospital, gender, position, and workplace violence experience. One-on-one interviews have been conducted with the 14 respondents by two researchers with interview experience, respectively. The interview topic mainly includes the following content: (1) Understanding the work content and environment of psychiatric nurses; (2) The influencing factors of workplace violence; (3) The needs and attitudes of psychiatric nurses towards violence prevention; (4) Problems in preventing workplace violence; and (5) Opinions on the “ideal” violence prevention program. All interviews have been audio-recorded and then transcribed verbatim. Qualitative data from one-on-one interviews have been analyzed using qualitative methods of thematic analysis. To define and compare all primary and secondary themes, two researchers independently have read and analyzed these transcripts. After summary in the text and tables, interpretation and discussion with the co- researchers to obtain the opinions of the co-investigators resulted in a broader list of attributes.

#### Focus-group discussions

The focus group discussion has been not part of the qualitative study, but rather a preparatory phase for designing the DCE. Three focus discussion groups, each consisting of three experts/leaders and three psychiatric nurses, have been organized to discuss potential attributes obtained from qualitative interviews and literature review and assign corresponding levels to them. In the focus group discussion, leaders/experts believe that the attributes “attention of leaders “and “coordination between the superior and subordinate” may affect each other. The higher the level of attention a leader places, the better the coordination between superiors and subordinates will be. Therefore, the attribute “coordination between the superior and subordinate” has been removed. At the same time, psychiatric nurses believe that security guard are very important in preventing workplace violence, so the level of attribute “collaboration “should include the collaboration of security guards, nurses and doctors, so as to reflect the supporting role of hospital security in preventing violence. Nurses believe that scheduling and collocation is very important, and reasonable collocation can help to prevent workplace violence to a certain extent, so the attribute “scheduling” has been added. Thereafter, all attributes have been discussed in focus groups, and the final six most important attributes have been selected and levels have been assigned to them. The final attribute levels are shown in Table 1.

**Table 1:** List of attributes and levels.

Attributes	Levels	Description
Emergency drills for violent response	As required	Emergency drills are carried out according to specific circumstances or the needs of nurses.
	Regular	The frequency of emergency drills is fixed, such as once every month.
Collaboration	Comprehensive	In addition to the collaboration between doctors and nurses, security also provides strong support.
	Middle	Mainly doctors and nurses collaborate, with almost no support from security.
	Scarce	Without support from security and doctor, it was almost the nurses who dealt with the violence on their own.
Violence prevention tools	Abundant	The variety and quantity of tools for preventing violence are complete and sufficient.
	Medium	The variety and quantity of tools to prevent violence are modest.
	None	There are no tools for preventing violence.
Attention from leaders	Great	Leaders attach great importance to violence prevention, often discussing possible prevention plans with nurses, and actively communicating with patients and their families to reduce the occurrence of violence
	General	Leaders place average importance on violence prevention, occasionally discussing possible prevention plans with nurses, and occasionally communicating with patients and family members
	Little	Leaders do not attach importance to violence prevention, believing that these are inevitable and rarely discussing with nurses and possible prevention plans
Scheduling	Relatively fixed	The partners of nurses in each shift are relatively fixed and rarely change
	Unfixed	The partners of nurses in each shift are random and not fixed
Involvement of family members of patients	Yes	Family members of patients participate in violence prevention, such as restraining and admonishing patients.
	No	Family members of patients do not participate in violence prevention.

Ten nurses who did not participate in one-on-one interviews and focus group discussions have been provided with a list of attribute levels determined. They were asked to “think-aloud” for each attribute level (45). The purpose of this exercise is to explore more potential attribute levels of DCE, which motivates psychiatric nurses to think about more factors that may affect their prevention of workplace violence. Then, these 10 psychiatric nurses have been interviewed via face-to-face or telephone interviews to ask for their views on attributes and levels, and their feedback has been recorded. They feel that these attribute levels are appropriate and reflect their concerns in violence prevention. In addition, they have suggested a description of the definitions at each level, which would be more helpful for understanding. The attribute level list has been revised based on their feedback and used in choice sets generation.

### Generating choice sets

**Figure 1:**
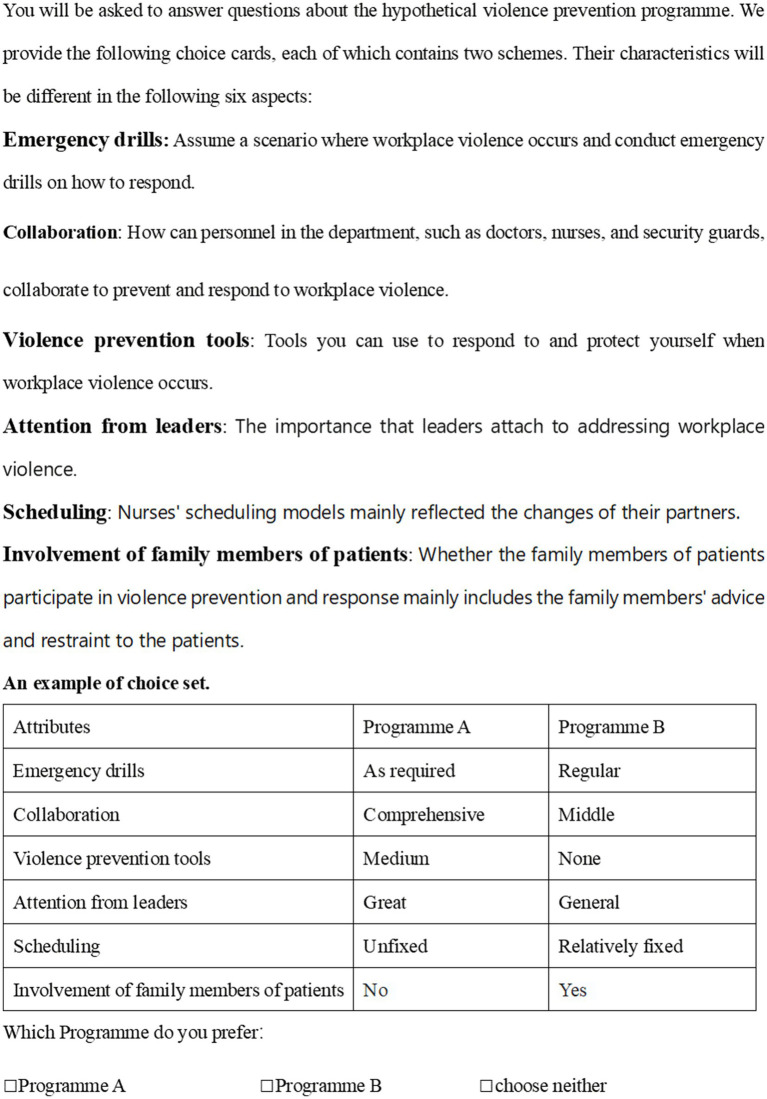
An example of choice set.

A full factorial design that includes all possible combinations is considered the most ideal method, as all interaction effects can be studied. However, full factorial designs can generate too many choice sets, consume too much time and financial resources in actual surveys, and produce too high a cognitive burden on respondents. Therefore, prior parameter values have been specified for each fixed parameter, and Ngene software has been used to generate a D-efficient design limiting the number of choice sets to 36, which has been randomly divided into three blacks, each containing 12 choice sets. The example of a choice set is showed in Figure 1.

### Questionnaire design and pilot testing

The questionnaire consists of three parts, namely the study introduction, the general information questionnaire, and the DCE choice tasks:

Study introduction: In this part, the purpose of the study and the precautions for completing the questionnaire are introduced, and the informed consent form has been provided to the respondents.

General information questionnaire: This part mainly includes social Demographics characteristics, such as gender, age, seniority, position, income, education level and workplace violence experience, to explore how this information may affect preferences.

Before providing psychiatric nurses with choice tasks, the attributes and levels of violence prevention programs are described to ensure that participants are clear. In addition to the 12 choice sets, the fourth choice set has been repeatedly included as the thirteenth choice set (not include in statistical analysis) to test response consistency. In each choice set, in addition to the two alternatives, an opt-out option (neither option is preferred) has been included to understand what participants would choose if they were obliged (forced to choose).

Pilot testing: This section includes participants filling out DCE questionnaires and conducting personal cognitive interviews. To date, there are no consistent guidelines on how to determine sample sizes for pilot testing in DCE. To ensure sufficient heterogeneity, we have referred to pilot testing samples designed by other DCEs (*N* = 6–24) (46–48) and ultimately surveyed 20 nurses who are asked to ‘think-aloud’ during the completion of each choice set. After completing the DCE choice tasks, interviews have been conducted with participants participating in the pilot testing to further refine the wording of DCE and improve its comprehensibility. Participants have responded that the questionnaire is appropriate in length and easy to understand. At the same time, based on their suggestions, we have added an example of choice set to facilitate understanding. A preliminary analysis of these 20 questionnaires has been performed to verify prior information, and the results have been used as input for another run using Ngene software to optimize choice set generation.

### Sampling and recruitment

The target population of this study is psychiatric nurses. Inclusion criteria: (1) Psychiatric nurses working in hospitals; and (2) Participate in direct patient care. Exclusion criteria: (1) not on duty during the study period (such as asking for leave, vacation or going out to study); (2) nursing students; and (3) nurses who did not want to participate in this study.

There is no unified guideline to determine the sample size of DCE. Pearmain believed that a discrete choice experiment could be carried out smoothly with 100 samples (49); Lancsar and Louviere (41) indicated that the determination of sample size in discrete choice experiment should be based on the number of questionnaire versions, and the sample size of each version should not be less than 20 to meet the statistical requirements. The rule of thumb proposed by Johnson and Orme is the most widely used. It considers the main effect model of DCE, and believes that the sample size of a DCE depends on the maximum number of attributes and its levels, the number of choice sets and the number of alternatives in each choice set, that is N > 500c/(t × a) (50, 51). Where N represents the sample size, 500 is a fixed variable, *c* represents the maximum number of levels of any attribute, *t* represents the number of choice sets in each questionnaire (excluding the repeatedly included choice sets), and *a* represents the number of alternatives in each choice set (because the “opt-out item” has no attributes and levels, it is not included). In our study, *t* = 12, *C* = 3, *a* = 2, and the number of samples for each version is calculated according to this formula to be 63.

This study contains three versions of the DCE choice tasks, and the number of samples required is 237, taking into account 20% of invalid questionnaires. Therefore, we plan to distribute 80 copies of each version. We plan to conduct convenient sampling nationwide to obtain the WeChat or email addresses of nurses who meet the inclusion and exclusion criteria, and send them an electronic version of the questionnaire through WeChat or email. The questionnaires have received by the respondents are randomized. The data collection for this study will begin in July 2024 and will be completed by June 2025.

### Analysis plan

SPSS 24.0 will be used for descriptive analysis of social Demography, and Stata 16.0 will be used for analysis of DCE data (52). Multiple logit models (MNL) have the characteristics of low error rate, low technical maturity, and low sample requirements. Using an MNL model as a framework in the early stages will help optimize the overall model, including finding more explanatory variables and making the level of factors more reasonable. Therefore, we will use an MNL to explore the trade-offs between the characteristics of violence prevention measures included in the choice task. Analyzing the preferences of psychiatric nurses for prevention of workplace violence will help explore which attributes (and levels) influence psychiatric nurses’ choice of violence prevention programs. According to their choice, the importance of these attributes (and levels) and their interaction with demographically relevant characteristics of nurses such as gender, education level, and age will be determined.

**Figure 2:**
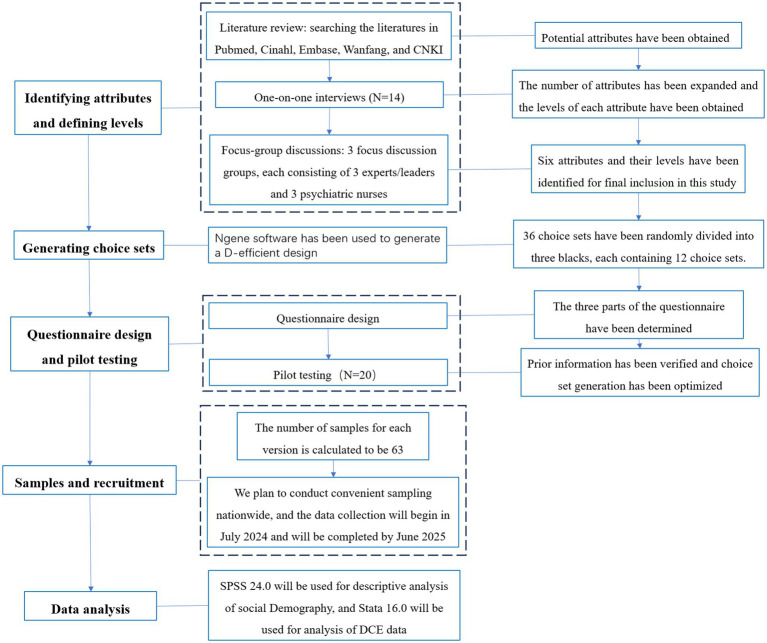
The development process of the DCE.

The MNL model cannot handle random preference differences and ignores individual heterogeneity. The mixed logit model, which accounts for heterogeneity in individual preferences, is the current standard and assumes that all attributes and alternative specific constants are random normally distributed. Therefore, a mixed logit model will be estimated, regressing each parameter with each sociodemographic characteristic interaction in turn, and the relative importance of each attribute for patient choice can be derived from the model by exploring the estimated parameters and their standard errors. We plan to choose some arbitrarily defined base violence prevention program and will use the nlcom command to evaluate the probability of an alternative violence prevention program. That is, when the level of one or more attributes changes compared to the base violence prevention program, then how the probability of a nurse receiving a violence prevention program will change. The development process of the DCE is showed in Figure 2.

## Limitation

There are some limitations in our study. Firstly, considering that too many attributes and levels can impose a cognitive burden on respondents, only the six most important attribute levels were included in our study. But it should be recognized that the excluded attributes may also be important, which may limit our discussion of the results in the future. Secondly, our study is a DCE, which explores the stated preferences of the respondents, and the degree of consistency between stated preferences and revealed preferences cannot be verifed.

## Ethics statement

This study was approved by the Ethics Committee of People's Hospital of Deyang city (the ethics number: DY20230701).

## Author contributions

PX: Writing – original draft, Writing – review & editing, Methodology. H-qL: Writing – original draft, Writing – review & editing. LT: Data curation, Supervision, Writing – review & editing. HY: Data curation, Investigation, Writing – review & editing.

## References

[1] National Institute for Occupational Safety and Health: Occupational violence; (2018) Available at: https://www.cdc.gov/niosh/topics/violence/default.html

[2] WHO. Joint programme on workplace violence in the health sector. (2021) Available at: https://www.who.int/violence_injury_prevention/injury/work9/en/.(Accessed June 5, 2020).

[3] Odes R Chapman S Harrison R Ackerman S Hong O. Frequency of violence towards healthcare workers in the United States' inpatient psychiatric hospitals: A systematic review of literature. Int J Ment Health Nurs. (2021) 30:27–46. doi: 10.1111/inm.12812, PMID: 33150644

[4] Dean L Butler A Cuddigan J. The impact of workplace violence toward psychiatric mental health nurses: identifying the facilitators and barriers to supportive resources. J Am Psychiatr Nurses Assoc. (2021) 27:189–202. doi: 10.1177/10783903211010945, PMID: 33998326

[5] Lu L Lok KI Zhang L Hu A Ungvari GS Bressington DT . Prevalence of verbal and physical workplace violence against nurses in psychiatric hospitals in China. Arch Psychiatr Nurs. (2019) 33:68–72. doi: 10.1016/j.apnu.2019.07.002, PMID: 31711597

[6] Whiting D Lichtenstein P Fazel S. Violence and mental disorders: a structured review of associations by individual diagnoses, risk factors, and risk assessment. Lancet Psychiatry. (2021) 8:150–61. doi: 10.1016/s2215-0366(20)30262-5 33096045

[7] Iozzino L Ferrari C Large M Nielssen O de Girolamo G. Prevalence and risk factors of violence by psychiatric acute inpatients: A systematic review and Meta-analysis. PLoS One. (2015) 10:e0128536. doi: 10.1371/journal.pone.0128536, PMID: 26061796 PMC4464653

[8] Sim I Ahn K Hwang E. Experiences of psychiatric nurses who Care for Patients with physical and psychological violence: a phenomenological study. Int J Environ Res Public Health. (2020) 17:159. doi: 10.3390/ijerph17145159, PMID: 32708899 PMC7400158

[9] Kobayashi Y Oe M Ishida T Matsuoka M Chiba H Uchimura N. Workplace violence and its effects on burnout and secondary traumatic stress among mental healthcare nurses in Japan. Int J Environ Res Public Health. (2020) 17:747. doi: 10.3390/ijerph17082747, PMID: 32316142 PMC7215457

[10] Liu X Yang H Hu Y Zhou Y Wang J Dong L . Incidence of workplace violence against nurses among Chinese hospitals: A meta-analysis. J Nurs Manag. (2022) 30:1490–501. doi: 10.1111/jonm.13427 34291858

[11] Abu Khait A Hamaideh S Aldalaykeh M. Psychiatric nurses' experiences and the emotional and psychological sequelae after being psychologically or physically assaulted in psychiatric units: A phenomenological study. Arch Psychiatr Nurs. (2022) 40:115–23. doi: 10.1016/j.apnu.2022.06.005, PMID: 36064234

[12] Fan S An W Zeng L Liu J Tang S Chen J . Rethinking "zero tolerance": A moderated mediation model of mental resilience and coping strategies in workplace violence and nurses' mental health. J Nurs Scholarsh. (2022) 54:501–12. doi: 10.1111/jnu.12753, PMID: 34866319

[13] Rasool S Wang M Zhang Y Samma M. Sustainable work performance: the roles of workplace violence and occupational stress. Int J Environ Res Public Health. (2020) 17:912. doi: 10.3390/ijerph17030912, PMID: 32024195 PMC7037902

[14] Shi L Wang L Jia X Li Z Mu H Liu X . Prevalence and correlates of symptoms of post-traumatic stress disorder among Chinese healthcare workers exposed to physical violence: a cross-sectional study. BMJ Open. (2017) 7:e016810. doi: 10.1136/bmjopen-2017-016810, PMID: 28765135 PMC5642665

[15] Global, regional, and national incidence, prevalence, and years lived with disability for 354 diseases and injuries for 195 countries and territories, 1990-2017: a systematic analysis for the global burden of disease study 2017. Lancet Oncol. (2018) 392:1789–858. doi: 10.1016/s0140-6736(18)32279-7, PMID: 30496104 PMC6227754

[16] Patel V Saxena S Lund C Thornicroft G Baingana F Bolton P . The lancet commission on global mental health and sustainable development. Lancet Oncol. (2018) 392:1553–98. doi: 10.1016/s0140-6736(18)31612-x 30314863

[17] Huang Y Wang Y Wang H Liu Z Yu X Yan J . Prevalence of mental disorders in China: a cross-sectional epidemiological study. Lancet Psychiatry. (2019) 6:211–24. doi: 10.1016/S2215-0366(18)30511-X 30792114

[18] Qin X Hsieh C. Understanding and addressing the treatment gap in mental healthcare: economic perspectives and evidence from China. Inquiry. (2020) 57:46958020950566. doi: 10.1177/0046958020950566, PMID: 32964754 PMC7517998

[19] China TNHCoPsRo. China Health Statistical Yearbook. (2020) Available at: https://www.yearbookchina.com/navibooklistn3020013080-1.html. (Accessed June 5, 2020).

[20] WHO. Mental health atlas Geneva. Switzerland: WHO (2017).

[21] Choi SH Lee H. Workplace violence against nurses in Korea and its impact on professional quality of life and turnover intention. J Nurs Manag. (2017) 25:508–18. doi: 10.1111/jonm.12488, PMID: 28547784

[22] Duan X Ni X Shi L Zhang L Ye Y Mu H . The impact of workplace violence on job satisfaction, job burnout, and turnover intention: the mediating role of social support. Health Quality Life Outcomes. (2019) 17:93. doi: 10.1186/s12955-019-1164-3, PMID: 31146735 PMC6543560

[23] Lim M Jeffree M Saupin S Giloi N Lukman K. Workplace violence in healthcare settings: the risk factors, implications and collaborative preventive measures. Annal Med Surg. (2022) 78:103727. doi: 10.1016/j.amsu.2022.103727, PMID: 35734684 PMC9206999

[24] Geoffrion S Hills D Ross H Pich J Hill A Dalsbø T . Education and training for preventing and minimizing workplace aggression directed toward healthcare workers. Cochrane Database Syst Rev. (2020) 2020:CD011860. doi: 10.1002/14651858.CD011860.pub2, PMID: 32898304 PMC8094156

[25] Rakatansky H. Workplace violence in hospitals and measures to address it. R I Med J. (2017) 100:11–2. PMID: 28564661

[26] Öztaş İ Yava A Koyuncu A. Exposure of emergency nurses to workplace violence and their coping strategies: A cross-sectional design. J Emerg Nurs. (2023) 49:441–9. doi: 10.1016/j.jen.2022.09.002 36307253

[27] López-Ros P López-López R Pina D Puente-López E. User violence prevention and intervention measures to minimize and prevent aggression towards health care workers: A systematic review. Heliyon. (2023) 9:e19495. doi: 10.1016/j.heliyon.2023.e19495, PMID: 37809629 PMC10558594

[28] Banerjee S Macdougall DW. Interventions to address and prevent violence toward health Care Workers in the Emergency Department. Canad J Health Technol. (2021) 1:88. doi: 10.51731/cjht.2021.88 36170468

[29] Iyus Y Ai M Hendrawati H Sri H. Interventions for reducing negative impacts of workplace violence among health workers: a scoping review. J Multidiscip Healthc. (2023) 16:1409–21. doi: 10.2147/jmdh.S412754, PMID: 37251104 PMC10216865

[30] Yubing H Qianqian L Rui L Min Z Yuming W Peipei S . Anti-violence measures developed by ILO and WHO: analysis of the prevalence of workplace violence and the effects of implementation in a general hospital in China. Front Public Health. (2023) 10:1049832. doi: 10.3389/fpubh.2022.1049832, PMID: 36589930 PMC9794770

[31] Yusheng T Yuchen Y Jianjian W Ting L Yamin L Jiansong Z. Workplace violence against hospital healthcare workers in China: a national WeChat-based survey. BMC Public Health. (2020) 20:582. doi: 10.1186/s12889-020-08708-3, PMID: 32349727 PMC7189471

[32] Xin Z Yizhi L Chunsheng Y Guan J. Trends in workplace violence involving health Care professionals in China from 2000 to 2020: A review. Med Sci Monit. (2021) 27:e928393–1. doi: 10.12659/msm.928393, PMID: 33417590 PMC7802374

[33] Yu X Ting-Ting C Shao-Yi Z Ling Z Na D Chun-Ya L . Workplace violence against Chinese health professionals 2013-2021: A study of national criminal judgment documents. Front Public Health. (2022) 10:1030035. doi: 10.3389/fpubh.2022.1030035, PMID: 36339236 PMC9627169

[34] Hiebert BJ Care WD Udod SA Waddell CM. Psychiatric Nurses' lived experiences of workplace violence in acute Care psychiatric units in Western Canada. Issues Ment Health Nurs. (2022) 43:146–53. doi: 10.1080/01612840.2021.1956656, PMID: 34379570

[35] Hong ST. Increasing violent attacks against physicians and healthcare workers are threats to the Korean society. J Korean Med Sci. (2019) 34:1–2. doi: 10.3346/jkms.2019.34.e13 PMC6318443 30618518

[36] Lozano JMG Ramón JPM Rodríguez FMM. Doctors and nurses: A systematic review of the risk and protective factors in workplace violence and burnout. Int J Environ Res Public Health. (2021) 18:3280. doi: 10.3390/ijerph18063280 33810020 PMC8004742

[37] Lancaste KJ. A new approach to consumer theory. Polit Econ. (1966) 74:132–57. doi: 10.1086/259131

[38] de Bekker-Grob E. Discrete choice experiment in health care:theory and applications. Rrotterdam: Erasmus University (2009).

[39] Mcfadden D. Conditional logit analysis of qualitative choice behavior. Frontiers in Econometrics. (1974).

[40] Johnson FR Lancsar E Marshall D Kilambi V Mühlbacher A Regier DA . Constructing experimental designs for discrete choice experiments: report of the ISPOR conjoint analysis experimental design good research practices TaskForce. Value Health. (2013) 16:13–3. 10.1016/j.jval.2012.08.2223 23337210

[41] Lancsar E Louviere J. Conducting discrete choice experiments to inform healthcare decision making: a user’s guide. Pharmacoeconomics. (2008) 26:661–77. doi: 10.2165/00019053-200826080-00004 18620460

[42] de Bekker-Grob E Ryan M Gerard K. Discrete choice experiments in health economics: a review of the literature. Health Econ. (2012) 21:145–72. doi: 10.1002/hec.1697 22223558

[43] Soekhai V Bekker-Grob E Ellis A Vass C. Discrete choice experiments in health economics: past, present and future. Pharmacoeconomics. (2019) 37:201–26. doi: 10.1007/s40273-018-0734-2, PMID: 30392040 PMC6386055

[44] Clark M Determann D Petrou S Moro D de Bekker-Grob E. Discrete choice experiments in health economics: a review of the literature. Pharmacoeconomics. (2014) 32:883–902. doi: 10.1007/s40273-014-0170-x, PMID: 25005924

[45] Ericsson KA Simon HA. Verbal reports as data. Psychol Rev. (1980) 87:215–51. doi: 10.1037/0033-295X.87.3.215

[46] Peng X Hui-Qin L Xia H. Whether preferences of gastric cancer patients after surgery for follow-up change over time? Analysis based on discrete choice experiment. Support Care Cancer. (2023) 31:234. doi: 10.1007/s00520-023-07699-2, PMID: 36964800

[47] Li H Xue H Yuan H Wan G Zhang X. Preferences of first-degree relatives of gastric cancer patients for gastric cancer screening: a discrete choice experiment. BMC Cancer. (2021) 21:959. doi: 10.1186/s12885-021-08677-9, PMID: 34445987 PMC8393792

[48] Li H Liu S Xue H Yuan H Zhang X. The Public's preferences for psychological interventions during the COVID-19 pandemic: A discrete choice experiment. Front Psych. (2022) 13:805512. doi: 10.3389/fpsyt.2022.805512, PMID: 35573350 PMC9091726

[49] Pearmain D Kroes EP. Stated preference techniques: A guide to practice. DEN HAAG: *Mode Choice* (1990).

[50] Orme BK. Getting Started With Conjoint Analysis: Strategies for Product Design and Pricing Research 2nd Edn. Madison, WI: Research Publishers. (2005).

[51] Johnson R. Orme B Getting the most from CBC. *Sequim: Sawtooth Research Paper Series, Sawtooth Software* (2003).

[52] Ye F Lord D. Comparing three commonly used crash severity models on sample size requirements: multinomial logit, ordered probit and mixed logit models. Anal Methods Accid Res. (2014) 1:72–85. doi: 10.1016/j.amar.2013.03.001

